# Psychosis, Catatonia and Tachypnoea Following Paliperidone Palmitate 3‐Monthly Injection Error: Case Report

**DOI:** 10.1155/crps/4635562

**Published:** 2026-08-18

**Authors:** Milla R. McLean, Lee Hanley, M. J. Yoo

**Affiliations:** ^1^ Department of Psychiatry, The Royal Melbourne Hospital, Melbourne, Victoria, Australia, thermh.org.au; ^2^ Department of Microbiology and Immunology, The University of Melbourne, Melbourne, Australia, unimelb.edu.au; ^3^ Department of Psychiatry, Melbourne Medical School, The University of Melbourne, Melbourne, Australia, unimelb.edu.au

**Keywords:** anti-psychotic, case report, long-acting injection, medication error, paliperidone palmitate

## Abstract

**Introduction:**

Paliperidone palmitate 3‐monthly (PP3M) is a long‐acting Injection (LAI) formulation of the anti‐psychotic medication paliperidone, also known as 9‐hydroxyrisperidone (9‐OH‐RIS). There is a notable absence of clinical guidelines or case reports pertaining to PP3M overdose and management of incorrect dosing.

**Patient Concerns and Clinical Findings:**

We present the first reported case of PP3M administered at 1 monthly intervals for three consecutive months in the community setting and describe the inpatient management of psychosis and catatonia occurring in the context of this overdose.

**Diagnoses, Interventions and Outcomes:**

During this patient’s admission to an inpatient psychiatric unit, our team, involving specialised pharmacy, navigated the acute management of psychosis in the context of PP3M overdose in the absence of published guidelines. We share the use of electroconvulsive therapy (ECT) and oral lorazepam in managing this patient’s acute psychosis and catatonia presentation and provide a rationale for the timing of safe recommencement of paliperidone oral medication. The patient was safely discharged home.

**Conclusions:**

We highlight the need for LAI overdose guidelines as an important area for review and development of clinical guidelines as we see increasing use of paliperidone palmitate 1‐monthly (PP1M), PP3M, and paliperidone palmitate 6‐monthly injection (PP6M) in the community.

## 1. Introduction

Paliperidone palmitate is a long‐acting injection (LAI) formulation of the second‐generation anti‐psychotic medication paliperidone, also known as 9‐hydroxyrisperidone (9‐OH‐RIS), used to treat psychotic illnesses such as schizophrenia [1]. In Australia, paliperidone palmitate LAI is available in three different modified‐release formulations differentiated by their frequency of administration—paliperidone palmitate 1‐monthly injection (PP1M), paliperidone palmitate 3‐monthly injection (PP3M) and paliperidone palmitate 6‐monthly injection (PP6M). LAI formulations offer advantages for both patients and clinicians, including stable serum drug concentrations and consistent treatment in preventing relapse [2].

Studies suggest that the clinical efficacy of paliperidone palmitate is highest in those with 70%–80% dopamine receptor D2 (D2R) occupancy, with adverse effects occurring at greater than 80% D2R occupancy [3, 4]. LAIs formulations of 9‐OH‐RIS may allow for serum concentrations to be stable such that optimal D2R occupancy is achieved; however, they pose challenges in overdose because clinical signs of toxicity may be unpredictable or prolonged. In this context, LAIs require careful administration due to their irreversibility once administered. There is currently limited literature regarding paliperidone palmitate LAI toxicity and its management.

Jin and Malik [5] describe a case of iatrogenic PP1M overdose in an elderly patient and found no adverse cardiac/circulatory nor neurological complications from the overdose. Parker et al. [6] describe a case of PP3M overdose in a patient with schizophrenia whose clinical state remained unchanged in the context of the overdose. Ojimba et al. [7] postulate that a patient who was administered two PP1M injections in error whilst being established on PP1M did not experience side effects due to the possibility of being a fast metaboliser. Significant adverse effects of high serum paliperidone published through case reports include acute dystonia from oral paliperidone overdose [8] and death from circulatory failure and multi‐organ failure in a patient who received PP1M [9]. Several of these case reports utilise plasma therapeutic drug monitoring to inform their clinical and treatment decision‐making [5, 6, 9].

We present the first reported case of PP3M administered at 1‐monthly intervals for three consecutive months found in a patient with acute psychosis and catatonia. Through this case, we illustrate the role of electroconvulsive therapy (ECT) in the treatment of psychosis and catatonia with high serum 9‐OH‐RIS levels and contribute to the understanding of managing LAI overdose.

## 2. Patient Information

A 25‐year‐old man presented to our hospital emergency department (ED) with acute psychosis and catatonia. The history of presenting complaint included 3‐weeks worsening duration of auditory hallucinations, paranoia and persecutory delusions on a background of an established history of schizophrenia with catatonia that has been responsive to ECT in the past. Comorbid medical conditions included mild intellectual disability, hyperlipidaemia, insulin resistance and obesity (BMI 35). Past surgical history included appendicectomy and Achilles tendon repair.

## 3. Clinical Findings

On admission to hospital, the patient presented with paranoid delusions, auditory hallucinations and thought blocking. His main physical concern was tachypnoea with a respiratory rate up to 45 respirations per minute in the absence of hypoxia. There was no clinical evidence of malignant catatonia or neuroleptic malignant syndrome (NMS) as the patient was afebrile with no evidence of autonomic instability. There were no signs of extra pyramidal side effects (EPSE), including no acute dystonia, akathisia, parkinsonism or tardive dyskinesia. The Bush–Francis Catatonia Rating Score was 20 on day 2 of admission to the adult inpatient psychiatric unit.

The patient and family reported that regular medications in the community were paliperidone palmitate LAI and olanzapine 20 mg nocte. The medication regimen was initially documented by the ED pharmacist following confirmation with the general practice (GP) clinic to whom the patient was known, the patient, and the carer. However, the atypical dosing interval was not recognised as erroneous at this stage. Following transfer to the psychiatric unit, a specialist psychiatric pharmacist review identified that PP3M had been administered 1‐monthly, rather than the intended 3‐monthly interval. Administration of further doses were withheld following recognition of the error.

Previously, the patient was maintained on PP1M 75 mg, however, he had experienced relapse requiring psychiatric admission, and the dose was increased to PP1M 150 mg. Following two doses of PP1M 150 mg administered through a community‐based psychiatry clinic, the patient’s care was transferred to a GP. Under the care of the general practitioner across 3 months, the patient subsequently received three doses of PP3M 175 mg at 4‐week intervals. In Australia, PP3M prescribing is PBS Authority Required (streamlined) and indicated for patients adequately stabilised on PP1M for at least 4 months or following PP6M therapy; however, prescribing is not restricted to specialist psychiatric services. The most recent dose was administered 4 days prior to the patient’s presentation to ED. The cumulative dose of PP3M was 525 mg over a 70‐day period. Although the cumulative administered dose of PP3M 175 mg over 3 months numerically approximated the equivalent PP1M 150 mg monthly exposure described in the product information, administration of PP3M at 4‐week intervals represented a pharmacokinetic overdose due to overlapping prolonged release depot exposure. Given the extended half‐life and delayed peak serum concentrations of PP3M, repeated monthly administration likely resulted in cumulative paliperidone exposure exceeding the intended therapeutic levels. Open disclosure of the medication error was completed with the patient and his family during this admission and communication with the GP around the error was completed in verbal and written form in consultation with the hospital‐GP liaison service.

## 4. Diagnostic Assessment

**Figure 1:**
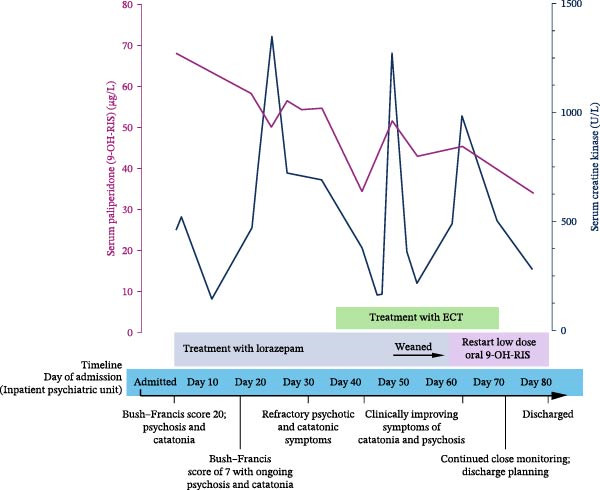
Clinical timeline and graphical representations of; serum creatine Kinase levels (dark blue line; U/L across *x*‐axis days of inpatient admission); serum paliperidone 9‐OH‐RIS levels (purple line; µg/L across *x*‐axis days of inpatient admission); Bush–Francis score (Bush–Francis Catatonia Rating Scale), serum Paliperidone (9‐OH‐RIS), electroconvulsive Therapy (ECT).

The serum 9‐OH‐RIS level at the time of admission was 68.2 µg/L (Figure 1). Paliperidone was withheld throughout most of the admission. The overall trajectory of serum 9‐OH‐RIS level subsequently trended down, with tri‐modal peaks on day 29 (56.6 µg/L), day 50 (51.8 µg/L) and day 64 (45.4 µg/L), respectively.

The patient demonstrated elevated levels of creatinine kinase (CK) (Figure 1). CK on admission was 526 U/L. The overall trajectory of CK trended down, with tri‐modal peaks on day 26 (1355 U/L), day 50 (2386 U/L) and day 64 (989U/L), respectively. The patient did not experience any acute or chronic renal dysfunction with an estimated glomerular filtration rate (eGFR) above 90 mL/min/1.73 m^2^ across the entirety of the admission with no evidence suggestive of rhabdomyolysis. NMS was monitored for across the admission and excluded in that the patient did not meet Leveson diagnostic criteria. They had no lead‐pipe rigidity, no episodes of hyperthermia, no autonomic instability, no episodes of tachycardia, no leucocytosis and no evidence of metabolic acidosis. QTc was monitored throughout the admission with no evidence of prolongation.

**Figure 2:**
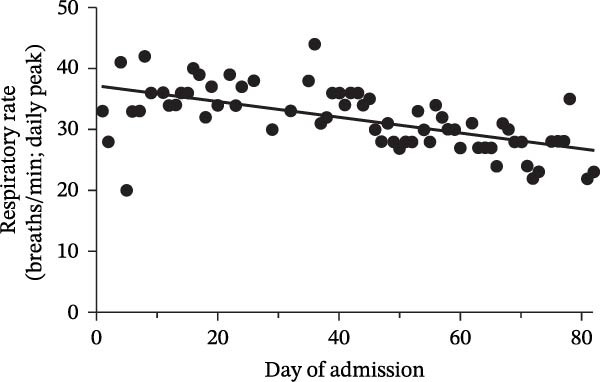
Respiratory rate measured as daily peak with linear regression line; measured in breaths/min across *x*‐axis days of inpatient psychiatric admission.

The patient demonstrated tachypnoea that waxed and waned. The initial impression from ED assessment attributed tachypnoea to obesity hypoventilation syndrome (OHS), considering the patient’s central obesity. However, OHS was later deemed less likely due to normal serum bicarbonate and the absence of hypercapnia on early‐morning arterial blood gas. CT chest identified lobar changes suggestive of asymptomatic inflammation, but antimicrobial treatment did not significantly affect the respiratory rate. Other investigations including autoantibody serology and ventilation‐perfusion scans were inconclusive and did not identify any cardiorespiratory cause for tachypnoea. The respiratory rate overall trended down throughout the admission (Figure 2).

## 5. Therapeutic Intervention

On admission, olanzapine was reduced from 20 to 5 mg/day due to concerns of possible antipsychotic overdose but to mitigate for risk of rebound psychosis with sudden cessation of antipsychotic medications. A blood sample was taken to obtain the serum 9‐OH‐RIS level, but the result of this was not available until 16 days later. To treat catatonia, lorazepam was commenced at 1–2 mg three times a day (maximum 6 mg/24 h). Unfortunately, the patient continued to experience distressing auditory hallucinations.

Accordingly, on day 3 of admission, olanzapine was cautiously increased to 10 mg daily, following close clinical monitoring that continued to demonstrate no features of NMS in the context of persistently elevated but down‐trending creatine kinase (CK) levels. However, no significant improvement in psychotic symptoms was observed following this dose increase.

Over the subsequent 27 days, the patient’s auditory hallucinations remained prominent and distressing. Olanzapine was therefore gradually further increased to 20 mg/24 h, consistent with the patient’s pre‐admission maintenance dose in the community. This decision was carefully balanced against the risk of recurrent NMS, with further dose increase limited by a rise in CK levels, which peaked at 1355 U/L on day 26. During this period, lorazepam was continued at varying doses. Multiple attempts were made to reduce the lorazepam dose during this time; however, each reduction was associated with the re‐emergence of catatonic features, necessitating ongoing treatment.

By day 30, both the patient’s psychotic symptoms and catatonia remained refractory to pharmacological management. Therefore, ECT was explored as a treatment option. The patient was assessed as lacking the capacity to provide informed consent for ECT. Consequently, a decision was made collaboratively between the treating team and the patient’s family, with consideration given to the patient’s preferences, to pursue ECT in the context of the patient’s continued suffering and lack of improvement. ECT was subsequently authorised by the Mental Health Tribunal (MHT).

The first ECT treatment was administered on day 36. Over the subsequent 6 weeks, the patient received a total of 12 bilateral ECT treatments. Bilateral ECT was selected over right unilateral ECT due to its greater efficacy in the treatment of catatonia. During this period, the patient demonstrated a gradual improvement in both auditory hallucinations and catatonic symptoms.

On day 63, the decision was made to recommence oral paliperidone at 3 mg daily. By this time, the patient had demonstrated marked and sustained improvement in mental state with ECT. During the admission, three peaks in creatine kinase (CK) and serum 9‐hydroxyrisperidone levels were observed. These fluctuations were hypothesised to correspond temporally with the three administrations of the PP3M formulation in the months preceding admission. Following resolution of the final peak and an overall down‐trending trajectory in CK levels, reinitiation of oral paliperidone was undertaken to facilitate an eventual transition back to a long‐acting injectable formulation.

This strategy was consistent with the patient’s prior stability on paliperidone palmitate and aimed to support a future return to a paliperidone palmitate LAI regimen, in alignment with the patient’s and family’s preferences.

## 6. Follow‐Up and Outcomes

The patient was discharged home from the ward on day 82 to the care of a community‐based psychiatry clinic with resolution of psychosis, catatonia and tachypnoea. The patient was discharged on the antipsychotic medications 3 mg paliperidone oral and 20 mg olanzapine oral with recommendation for weekly 9‐OH‐RIS monitoring and recommencement of PP1M 75 mg if clinically appropriate at the treating team’s discretion.

## 7. Discussion

We present the first reported case of PP3M administered at 1‐monthly intervals for three consecutive months. PP3M has a modified‐release profile with a reported half‐life of 84–139 days and peak serum concentration 30–33 days post‐injection [10]. However, in situations of overdose secondary to inappropriately frequent administration, the pharmacokinetics of PP3M are unestablished and difficult to predict. Our case study describes an unprecedented situation in which PP3M was administered at 1‐monthly intervals for three consecutive months. Serum 9‐OH‐RIS demonstrated an overall downward trajectory, with three up‐ticks (Figure 1). This tri‐modal pattern may reflect the three overlapping peaks of PP3M that were administered 1‐monthly, for 3 months prior to admission. This tri‐modal pattern in serum 9‐OH‐RIS was echoed in the tri‐modal peaks of serum CK (Figure 1).

Therapeutic ranges regarding serum 9‐OH‐RIS are not universally defined. Most sources recommend a range of 20–60 µg/L, with levels 40 µg/L and above reserved for treatment‐resistant illness due to the risk of EPSE [3]. Riedel et al. [11] suggest that at comparable oral doses of risperidone, for which 9‐OH‐RIS is the active moiety, those with lower serum 9‐OH‐RIS demonstrated an improved clinical response compared to those with higher serum 9‐OH‐RIS [11]. Due to inter‐individual and intra‐individual factors that may impact serum levels, the determination of a therapeutic range for serum 9‐OH‐RIS likely requires case‐by‐case consideration that is individualised to each patient [12]. We utilised serum 9‐OH‐RIS as part of the decision‐making process to guide the recommencement of oral paliperidone. We noted a clinical correlation between tachypnoea and serum 9‐OH‐RIS above 40 µg/L. Consultation with senior pharmacy, quaternary toxicology and pharmaceutical representatives for PP3M were unable to provide specific guidance on timing of paliperidone recommencement due to the unprecedented nature of the medication error. Therefore, in the absence of guidelines, an empirical clinical decision was made to consider recommencement of oral paliperidone once serum 9‐OH‐RIS was measured below 35 µg/L, acknowledging that time‐to‐results for drug serum levels can take several weeks and levels continued to fluctuate between 35–45 µg/L post recommencement. Levels continued to drop to <35 µg/L prior to discharge (Figure 1), and there were no evident adverse effects from recommencement of oral paliperidone and the patient had recovered from acute psychotic and catatonic symptoms.

The patient presented with psychosis, catatonia and tachypnoea, with elevated CK, and close inpatient monitoring occurred over subsequent weeks due to the need to monitor for delayed signs of toxicity [7]. These presenting features paradoxically suggest both a relapse of schizophrenia and paliperidone toxicity.

Our primary hypothesis is that this presentation predominantly reflects paliperidone toxicity related to excessive dopamine blockade, causing symptoms of blunting and catatonia that are correlated with high serum 9‐OH‐RIS levels. Supporting this hypothesis, catatonia has previously been described as a feature of paliperidone toxicity [13–16].

An additional explanatory mechanism is dopamine super‐sensitivity psychosis (DSP), whereby chronic D2 receptor antagonism may result in compensatory receptor upregulation and paradoxical psychosis despite ongoing antipsychotic exposure [17, 18]. Given this patient’s history of chronic antipsychotic treatment and recent escalation of paliperidone dose, DSP remains a biologically plausible contributing mechanism for the patient’s persistent auditory hallucinations despite supratherapeutic serum 9‐OH‐RIS concentrations. The patient’s improvement following ECT is consistent with previous reports describing ECT use in DSP [19]; however, ECT response in this context is non‐specific and may reflect improvement in catatonia, psychosis or DSP. A less likely explanation for the patient’s presentation is that the transition of PP1M to PP3M resulted in a period of sub‐therapeutic exposure precipitating psychosis and catatonia, however sustained supratherapeutic serum 9‐OH‐RIS makes this less probable.

The tri‐modal pattern of CK measured in this case (Figure 1) suggests a relationship with serum 9‐OH‐RIS in the absence of NMS or rhabdomyolysis and did not appear to correlate with clinical signs of catatonia. This may represent massive asymptomatic creatine kinase elevation as has been described with paliperidone in a case report by Grandioso et al. [20] or may represent the impact of serum paliperidone on tissue damage, further investigations into the relationship between CK and paliperidone are necessary.

In summary, this case report presents several complexities that occurred in the context of paliperidone palmitate LAI overdose secondary to medication error. We describe the first instance of PP3M overdose in the literature due to consecutive 1‐monthly administration—a clinical situation which has not previously been reported in the literature to date. We highlight the need for an increased evidence‐base to support future clinical decision‐making in instances of paliperidone palmitate LAI overdose, particularly in the absence of published guidelines.

## Funding

No funding was received for this manuscript. Open access publishing facilitated by The University of Melbourne, as part of the Wiley‐The University of Melbourne agreement via the Council of Australasian University Librarians.

## Consent

The patient and family provided written informed consent for this case report publication.

## Conflicts of Interest

The authors declare no conflicts of interest.

## Data Availability

The de‐identified data that support the findings of this study are available upon request from the corresponding author. The data are not publicly available due to privacy or ethical restrictions.
